# End-to-Side Neurorrhaphy as Schwann Cells Provider to Acellular Nerve Allograft and Its Suitable Application

**DOI:** 10.1371/journal.pone.0167507

**Published:** 2016-12-01

**Authors:** Hidekazu Yoshizawa, Daiki Senda, Yuhei Natori, Rica Tanaka, Hiroshi Mizuno, Ayato Hayashi

**Affiliations:** Department of Plastic and Reconstructive Surgery, Juntendo University School of Medicine, Tokyo, Japan; The University of Akron, UNITED STATES

## Abstract

Axonal regeneration relies on support from proliferating host Schwann cells (SCs), and previous studies on acellular nerve allografts (ANGs) suggest that axons can regenerate into ANGs within a limited distance. Numerous studies have demonstrated that the supplementation of ANGs with exogenous factors, such as cultured SCs, stem cells, and growth factors, promote nerve regeneration in ANGs. However, there are several problems associated with their utilization. In this study, we investigated whether end-to-side (ETS) neurorrhaphy, which is an axonal provider, could be useful as an SC provider to support axonal elongation in ANGs. We found that ETS neurorrhaphy effectively promoted SC migration into ANGs when an epineurium window combined with partial neurectomy was performed, and the effectiveness increased when it was applied bilaterally. When we transplanted ANGs containing migrated SCs via ETS neurorrhaphy (hybrid ANGs) to the nerve gap, hybrid ANGs increased the number of regenerated axons and facilitated rapid axonal elongation, particularly when ETS neurorrhaphy was applied to both edges of the graft. This approach may represent a novel application of ETS neurorrhaphy and lead to the development of hybrid ANGs, making ANGs more practical in a clinical setting.

## Introduction

Nerve autograft has been a standard treatment option for reconstructing peripheral nerve injuries or defects; however, it is associated with a limited supply of the donor nerve and the elimination of the donor nerve function [1, 2]. Therefore, identifying alternatives to the nerve autograft has been widely investigated, and multiple types of nerve conduits, including silicone tubes and synthetic biodegradable tubes, have been experimentally and clinically developed in the last few decades [3]. However, the clinical usage of silicone conduits has been limited to gaps which are less than 10 mm [3]. Absorbable conduits were also limited to be less than 10 mm in a rat model [3–5] and within 28 mm for sensory nerves in a clinical setting [4, 6, 7].

Recently, acellular nerve allografts (ANGs) have been demonstrated as promising substitutes for short nerve gaps [2, 7]. The clinical usage of ANGs provided superior results compared with that of conduits and showed meaningful functional recovery not only for sensory but also for mixed and motor nerves in gaps up to 50 mm [8]. However, the suitable usage of ANG remains controversial.

In contrast, extracellular matrix molecules (ECM) and growth factors secreted by Schwann cells (SCs) are required for axonal elongation [9–11]. We demonstrated the relationship between axonal regeneration and migration of SCs through ANGs utilizing transgenic mice expressing fluorescence in SCs and axons, and a highly intimate interaction, described as a “dance,” exists between regenerating axons and proliferating migrated SCs [12, 13]. Axonal regeneration generally requires proliferating SCs from the donor nerve [14]; therefore, some type of supplementation for ANGs is necessary, particularly for long nerve defect [15]. Numerous studies have demonstrated that the supplementation of exogenous factors, including cultured SCs, stem cells, and growth factors, promotes axon regeneration [16]. However, there are several problems associated with their use, such as the time-consuming process of cell culture, teratoma formation with the usage of stem cells, and continuous administration for growth factors [17–19]. Furthermore, ethical concerns and safety are involved for utilizing these viable cells. Therefore, alternative methods for supplementation are required for the practical usage of ANGs for long gaps.

End-to-side (ETS) neurorrhaphy has been used as an option for an axonal provider [20]. Because the source of the regenerated axons has been previously elucidated [21–28], we concluded that collateral sprouting could occur mainly in the sensory axons without damage to the donor nerve and some types of injuries, such as axotomy or a compressive stimulus, are required for further axonal regeneration including motor nerve regeneration [26]. Although its effect as an axonal provider in a clinical setting varies [29, 30], ETS neurorrhaphy may be more useful as an SC provider to support axonal elongation in ANGs.

We previously reported the characteristics of SCs migration into the nerve allograft; host SCs could rapidly migrate into ANG from the proximal and distal anastomosis of the graft [31]. As compared with the aforementioned supplementation of ANGs that includes applying cultured SCs or stem cells, there are no ethical or safety concerns associated with utilizing ETS neurorrhaphy; therefore, we could promptly develop this technique for clinical application. If we could supply sufficient autologous SCs through ETS neurorrhaphy, it would enable us to create hybrid ANGs; and the potential benefit of this technique could be extensive.

In this study, we investigated whether ETS neurorrhaphy could be a useful technique as an SC provider in ANGs *in vivo*.

## Material and Methods

### Animals

In this study, we used Nestin-green fluorescent protein (GFP) transgenic mice expressing GFP in their SCs when proliferated, Thy1-yellow fluorescent protein (YFP) 16 transgenic mice expressing YFP in all axons, and non-fluorescent C57BL/6 mice. The animal husbandry was conducted in accordance to standards and regulations provided by the National Institutes of Health Guide for Care and Use of Laboratory Animals. All experiments were conducted in accordance with protocols approved by the Division of Comparative Medicine at the Juntendo University School of Medicine. Surgery was performed under inhalation anesthesia using isoflurane and intraperitoneal injections of pentobarbital. All efforts were made to minimize suffering. Nestin-GFP and Thy1-YFP 16 mice were inbred on a C57BL/6 background. Mice (7–12 weeks old) weighing 22–30 g underwent sciatic nerve transection and grafting. Nestin-GFP mice were gifted by Dr. Yamaguchi (Tokyo University, Tokyo), which were used to identify the ANGs containing migrated SCs using *in vivo* imaging and immunohistochemistry [32]. Homozygous Thy1-YFP 16 mice (available from The Jackson Laboratory, strain B6.Cg-Tg(Thy1-YFP)16Jrs/J) were used to track the course of all regenerated axons in the end-to-end nerve graft model and to evaluate motor endplate reinnervation. All animals and their wellbeing were observed throughout the experimental period. At the endpoint of the experimental period after surgery for each experiment described, the mice were euthanized humanely by cervical dislocation.

### Preparation of cold preserved nerve allograft

Sciatic nerve allografts harvested from C57/BL6J mice were independently processed and decellularized using a method of cold preservation, as described previously [33]. In brief, harvested nerves were directly placed into sterile petri dishes containing VIASPAN (DuPont Merck Pharmaceutical Company) with penicillin G (200,000 U/L), regular insulin (40 U/L), and dexamethasone (16 mg/L). Nerves were then stored at 4°C for a period of 7 weeks prior to implantation, and the preservation solution was changed weekly [33].

### Surgical model to study SC migration

**Fig 1:**
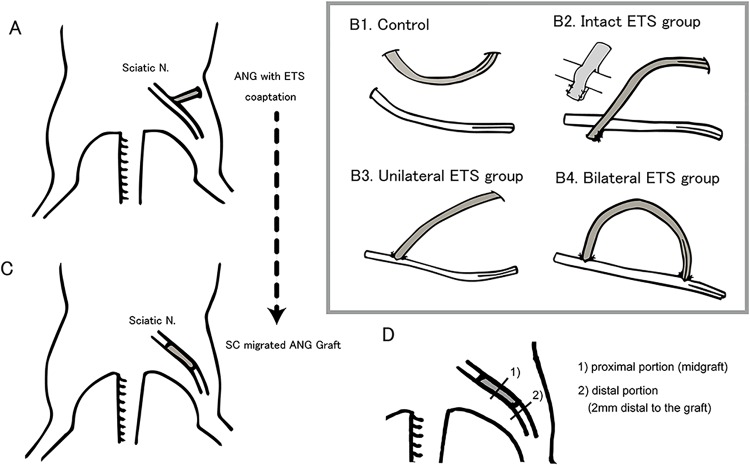
Schematic drawing of experimental model and design. A) Schematic drawing of the experimental model for Schwann cell (SC) migration ability. Cold preserved acellular nerve allografts (ANGs) were neurorrhapied to sciatic nerve with end-to-side (ETS) fashion. B) Schematic drawing of ETS neurorrhaphy for each group. B1) In the control group, the donor nerve was not in contact with the graft nerve. B2) In the intact ETS group, ETS neurorrhaphy was performed at one side of the graft by wrapping the donor nerve with the divided graft nerve. B3) In the unilateral ETS group, ETS neurorrhaphy was performed at one side of the graft by opening the epineurial window and by partial neurectomy. B4) In the bilateral ETS group, ETS neurorrhaphy was performed at both side of the graft with opening epineurial window and partial neurectomy. C) Schematic drawing of experimental model for the efficacy of ANGs containing migrated SCs. ANG containing migrated SCs (1 cm) was transplanted to the 5-mm nerve gaps of sciatic nerve in Thy-1 YFP mice. D) Schematic drawing of nerve cross section evaluation after engraftment.

All animals were anesthetized with the inhalation anesthesia technique of isoflurane and intraperitoneal injections of pentobarbital. First, a skin incision was made parallel to 2 mm posterior to the femur, and sciatic nerve was exposed with a microsurgical technique to include the sciatic notch proximally and its trifurcation to the tibial, peroneal, and sural nerves distally. A 1.0-cm cold preserved ANG from a C57BL/6J mouse was grafted to the sciatic nerves of a Nestin-GFP mouse via three types of ETS neurorrhaphy (Fig 1A). Four groups were made for this study. In group 1, the donor nerve was not in contact with the graft nerve and both edges of the graft nerve were intramuscularly embedded (control group) (Fig 1B1). In group 2, the donor nerve was neurorrhaphied ETS with a non-injury technique we described previously (intact ETS group) (Fig 1B2) [23]. In this model, one edge of the graft was slit 3 mm, and the resulting two sleeves wrapped around the donor sciatic nerve. This construct was secured to the donor sciatic nerve by suturing the sleeves to one another on the other side of the donor nerve without placing any sutures in the donor nerve itself and accomplished ETS coaptation atraumatically [26]. In group 3, the epineurium was removed and partial neurectomy of 1/5 degree was added at the suture site to the donor nerve, and ETS neurorrhaphy was then performed between one side of the graft and recipient nerve (unilateral ETS with partial neurectomy group) (Fig 1B3). In group 4, as in group 3, the epineurium was removed and a partial neurectomy of 1/5 degree was added to the donor nerve, and then both sides of the graft were neurorrhaphied with ETS (bilateral ETS with partial neurectomy group) (Fig 1B4). We serially imaged the nerve grafts at the time of surgery and 7, 14, 21, and 28 days after surgery using a fluorescence dissecting microscope (n = 4 per group). At 4 weeks after surgery, the animals were sacrificed and the grafts were harvested to analyze SC migration into the graft (n = 4 per group). Some of the graft samples were stored in 4% paraformaldehyde for histomorphometry and immunohistochemical analysis. The remaining graft samples were stored in RNAlater solution (QIAGEN, Valencia, CA) at −80°C, following which they were extracted at the 2- and 4-week endpoints for quantitative reverse transcriptase polymerase chain reaction (qRT-PCR) analysis (n = 5 per group).

### Surgical model for the efficacy of ANGs containing migrated SCs (hybrid ANGs)

A non-fluorescent 1.0-cm ANG from a C57/BL6J mouse was grafted to the sciatic nerves of a C57/BL6J mouse via two types of ETS neurorrhaphy with an aforementioned partial neurectomy group (Fig 1B3 and 1B4), and three groups were made for this study (group1, control; group2, unilateral ETS; and group3, bilateral ETS) (Fig 1B1, 1B3 and 1B4). After 4 weeks, 1 cm of the grafted ANG in which SCs migrated via each ETS neurorrhaphy was transplanted on to the 5 mm nerve gaps of sciatic nerves in Thy-1 YFP mice wherein all axons expressed YFP fluorescence. We attached the graft by suturing both edges on end-to-end neurorrhaphy with epineural sutures (n = 4 per group) (Fig 1C). In vivo imaging of regenerated YFP-labeled axons was conducted at the time of surgery and every 7 days (n = 4 per group). At 4 weeks after surgery, we evaluated axonal regeneration in the middle of the graft and distal to the graft under light and electron microscopes, and histomorphometry, such as evaluating total myelinated fiber count, g-ratio, myelin sheath thickness, and axon diameter, was performed (n = 5 per group). At 7 weeks after surgery, the reinnervated motor endplates were evaluated under a fluorescent dissecting microscope and the percentage of innervated endplate was calculated as described below (n = 4 per group).

### In vivo serial imaging and confocal imaging

We serially imaged the nerve grafts at the time of surgery and at 7, 14, 21, and 28 days later using a fluorescence dissecting microscope (MZ16FA Leica microsystem). To facilitate live imaging, mice were re-anesthetized and the grafted sciatic nerve was re-exposed and imaged with the fluorescent dissecting microscope for the exploration of SC migration and axonal elongation. To evaluate the distance of SC migration more accurately, SC migration from ETS coaptation was quantitatively assessed by preparing whole mount ANGs at 28 days after surgery. GFP-positive migrating cells in the entire graft could be observed using stack images from confocal microscopy.

The sciatic nerve graft was imaged with DFC300FX controlled by Leica Application Suite Advanced Fluorescence version 2.5 software (Leica microsystem) under GFP (488 nm) fluorescent and bright field filters. And the whole mount sciatic nerve graft was surveyed using TCS-SP5 (Leica microsystem). The images were recorded monochromatically using Leica Application Suite Advanced Fluorescence version 2.5 software (Leica microsystem). Images were standardized according to magnification (10×, 40×), exposure time (100–300 ms), orientation, brightness, and contrast.

### Immunohistochemistry

Harvested nerves were immunostained with S100 (1:1000, Dako, Carpinteria) for markers of SCs and NF-L(C-15) (1:50, Santa Cruz Biotechnology) for axon regeneration. Explanted nerves were fixed in 4% paraformaldehyde (Wako Pure Chemical Industries) at 4°C overnight and transferred to 30% sucrose for cryoprotection. Each graft was embedded in OCT Compound (Sakura Finetek Japan) and cut into 4-μm longitudinal sections using a cryostat.

To further confirm the SCs migration, grafts were stained for S100 and NF-L (C-15). Sections were then stained for Alexa 594-conjugated secondary antibodies (Molecular Probes). After antibody staining was complete, the slides were covered with VECTASHIELD mounting medium (Vector Laboratories).

### qRT-PCR

The gene expression of markers of SC migration (NRG1, ErbB2, S100) and senescence (p16^INK4A^) in ANGs was analyzed using qRT-PCR. Each sample was stored in RNAlater solution (QIAGEN) at −4°C overnight and then transferred to −80°C until RNA extraction. Reverse transcription was performed using a High-Capacity RNA-to-cDNA kit (Applied Biosystems). The transcription reaction was performed at 37°C for 2 h. The obtained cDNA was amplified using the reaction mixture of TaqMan FAST Universal PCR Master Mix (Applied Biosystems). All primers were purchased from Applied Biosystems and measured values were compared with glyceraldehyde-3-phosphate dehydrogenase as a house keeping gene [34]. The PCR mixtures were preincubated at 95°C for 20 s, followed by 40 cycles of 95°C for 3 s and 62°C for 30 s by ABI 7500 FAST (Applied Biosystems). The real-time data were analyzed so that the differences in gene expression levels between two different samples were calculated using the comparative delta crossover threshold (Ct) method [35].

### Histomorphometry and electron microscopy

Nerve cross sections at the midgraft (proximal section) and 2 mm distal to the graft (distal section) were evaluated 28 days after engraftment for regenerated axonal number, size, distribution, and g-ratios (Fig 1D). Images of the proximal and distal sections of the right sciatic nerves were digitized using a HT7700 microscope (HITACHI). For morphological evaluation, the procedure by de Medinaceli was used [36]. Briefly, a set of six images was obtained from each nerve portion, three random images from the periphery, and three random images from the center of the nerve to obtain a representative area per nerve segment [37]. The morphometric measurements were calculated in both large and small myelinated fibers. Morphometric measurements included the following: 1) average myelinated fiber diameter (μm); 2) average axon diameter (μm) of the myelinated fiber; 3) average myelin sheath thickness (μm); and 4) g-ratio (the quotient axon diameter/fiber diameter, a measurement of the degree of myelination)[38]. These morphological measurements were chosen to assess the difference between the models to study the efficacy of the ANGs containing migrated SCs.

### Motor endplate evaluation

The engrafted sciatic nerves and Extensor digitorum longus (EDL) muscles were harvested and fixed for 30 min. To label muscle fiber motor endplates, whole mounts of EDL muscles were soaked for 30 min in a 1:100 solution of Alexa 594-conjugated alpha-bungarotoxin (BTX; Molecular Probes). We calculated the total number of reinnervated endplates as a percentage of the total number of endplates 7 weeks following engraftment in Thy1-YFP16 mice. As described previously [39], the deep surface of BTX-labeled EDL muscle whole mounts were surveyed using TCS-SP5 (Leica microsystem) fluorescent microscopy at 10× and 20× to identify labeled terminal axons and endplates, and counted using a 40× objective and 488- and 568-nm lasers. At 7 weeks postoperatively, motor endplates captured within 25 randomly sampled areas of 100 μm^2^ were evaluated, and the number of innervated and denervated motor endplates was counted.

### Statistical analyses

Statistical analyses were performed using GraphPad Prism6 software (GraphPad software). One-way ANOVA was used to determine whether there are any statistically significant differences between the groups’ confocal imaging and histomorphometric data. In addition, differences between the qRT-PCR results within the groups were evaluated using two-way ANOVA. Histomorphometric data (serial in vivo imaging) was evaluated with a one-way repeated measures ANOVA. Newman–Keuls test was then performed to compare groups. Significance was established at p < 0.05. All results are reported as mean ± standard deviation.

## Results

### In vivo imaging of Schwann cell migration

**Fig 2:**
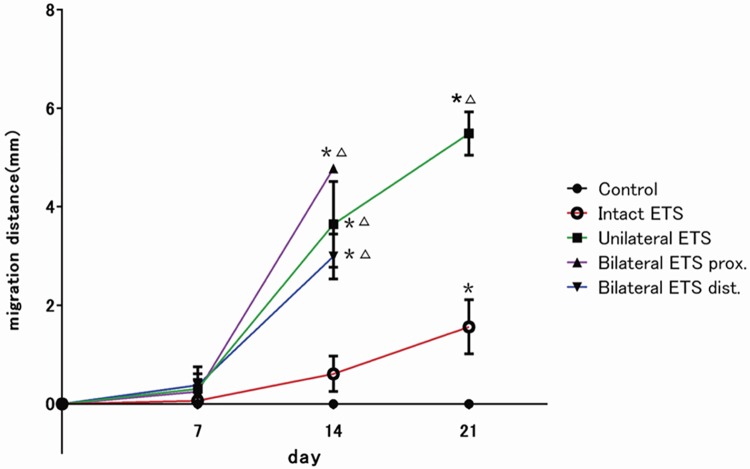
*In vivo* imaging of Schwann cell (SC) migration analysis. The mean migration distance from the edge of acellular nerve grafts were measured by *in vivo* imaging (n = 4 per group). The significant increase in SC migration was observed in all end-to-side (ETS) neurorrhaphy groups. 14 days after surgery. Partial neurectomy groups (the unilateral and bilateral ETS groups) affect long migration rather than intact ETS group. Differences between partial neurectomy and intact ETS group were significant. *Significantly different from control group (p < 0.05); Δ, significantly different from unilateral ETS group (p < 0.05).

By direct microscopic observation, ANGs appeared black, with no GFP expression by SCs at the time of engraftment. GFP-expressing cells continued to migrate throughout the graft from 7 to 28 days (Fig 2).

Seven days postoperatively, 0.06–0.305 mm of SC migration to ANGs was noted in all groups (except for the control group). The 14^th^ day postoperatively, 0.13 ± 0.59 mm of SC migration was noted in the intact ETS group, 3.64 ± 1.51 mm in the unilateral ETS group, 4.28 ± 0.35 mm in the bilateral ETS group at the proximal side, and 2.99 ± 0.46 mm in the bilateral ETS group at the distal side. We confirmed significant differences between the partial neurectomy ETS group (unilateral and bilateral ETS groups) and the control/intact ETS group (p < 0.05). On the 21^st^ day after surgery, 1.56 ± 0.78 mm and 5.49 ± 0.76 mm of SC migration was noted in the intact and unilateral ETS groups, respectively. In addition, SC infiltration of throughout a range of ANG from both sides was noted in the bilateral ETS group. We confirmed differences between the unilateral ETS group and the intact ETS group, intact ETS group and the control (p < 0.05). SC migration became progressive 14 days after surgery, and a difference of migration velocity (mm/week) was not apparent between the unilateral and bilateral ETS groups.

### Confocal imaging of SC migration

**Fig 3:**
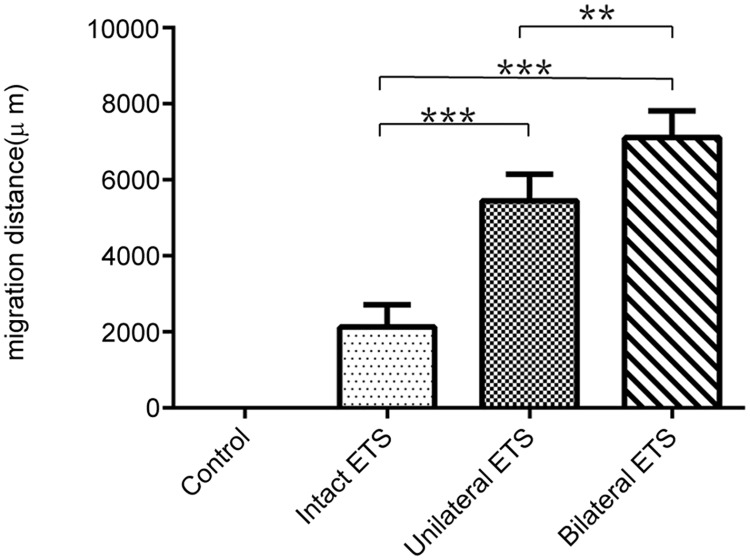
The distance of Schwann cell (SC) migration into the graft. The mean SC migration distance from edge of graft was assessed by confocal imaging of the whole mount graft (n = 4 per group). The bilateral end-to-side (ETS) group showed the longest distance compared with the other groups, followed by the unilateral ETS, intact ETS, and control groups. In addition, the control group did not show GFP-positive cells migrating into the graft. Differences between the control and the other groups were significant (p < 0.001). **p < 0.01, ***p < 0.001.

Whole mount imaging of the ANGs harvested at 28 days after surgery showed no migrating cells (0 μm) in the control group, whereas dramatically abundant migration of Nestin-GFP positive SCs was observed in the bilateral ETS group (7,111 ± 351 μm), followed by the unilateral ETS group (5,451 ± 351 μm) and intact ETS group (2,130 ± 337 μm) (Fig 3). Significant differences were confirmed between the control and the other groups (p < 0.001). The SCs migration distance of the bilateral ETS group was longer than that of the unilateral ETS group (p < 0.01) and the intact ETS group (p < 0.001).

### Confirmation of SCs migration by S100 and neurofilament antibody staining

**Fig 4:**
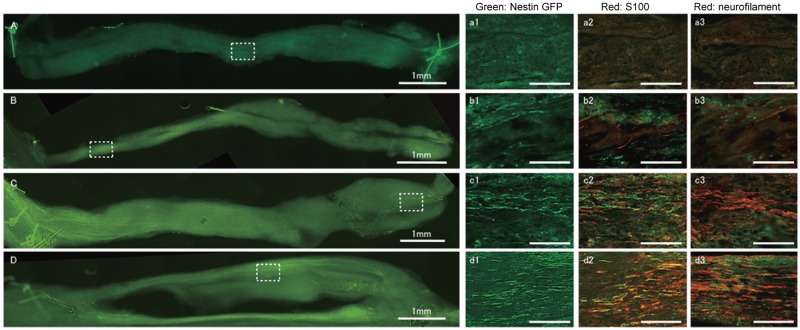
Confocal imaging and immunohistochemical staining of Schwann cell (SC) migration. A–D) Confocal image of acellular nerve allografts (ANGs) via four types of end-to-side (ETS) neurorrhaphies. Scale bar, 1 mm. a–d) Magnification of the inset outlined in A–D). a1–d1) Constitutive GFP expression of nestin-GFP mice. a2–d2) Anti-S100 antibody marked with Alexa 594-conjugated secondary antibody (Red: S100, Green: nestin-GFP), a3–d3) Anti-neurofilament antibody marked with Alexa 594 conjugated secondary antibody (Red: neurofilament, Green: nestin-GFP). A) In the control group, no migrated SCs and regenerated axons were observed. B) In the intact ETS group, a few SCs could migrate to nearby ETS coaptation; however, no regenerated axons were observed. C) In the unilateral ETS group, a small number of SCs migrated to the distal edge of the ANG, and a few regenerated axons were also confirmed at the distal edge of the graft. D) In the bilateral ETS group, a large number of migrated SCs and abundant regenerated axons were confirmed throughout the ANG. Scale bar, 100 μm.

To confirm our *in vivo* findings, the localization of SCs within ANGs was evaluated by immunohistochemical staining with anti-S100 antibody. Donor-derived SCs were double labeled with constitutively expressed GFP and anti-S100 antibody. The control group had no double-labeled migrating SCs (Fig 4a2). In the intact ETS group a few SCs migrated and remained at the ANGs nearby coaptation (Fig 4b2). We confirmed that a small number of SCs could migrate to the distal edge of the ANGs in the unilateral ETS group (Fig 4c2) and were further able to confirm a large number of migrated SCs throughout graft nerves in the bilateral ETS group (Fig 4d2).

Regarding neurofilament staining, abundant regenerated axons were colocalized with constitutively GFP-expressing SCs and anti-neurofilament antibodies in the bilateral ETS group as compared with the other groups (Fig 4a3–4d3).

### Differences in the expression of SC migration related markers after ETS grafting

qRT-PCR was used to further characterize the expression of SCs migration related markers in ANGs using ETS neurorrhaphy. ETS grafts were harvested 2 and 4 weeks postoperatively and were separated from the graft at the most proximal and distal suture lines. These tissue sections were then analyzed for the expression of SCs (S100), SCs migration related markers (NRG1 and ErbB2), and senescence or stress marker (p16^INK4a^).

**Fig 5:**
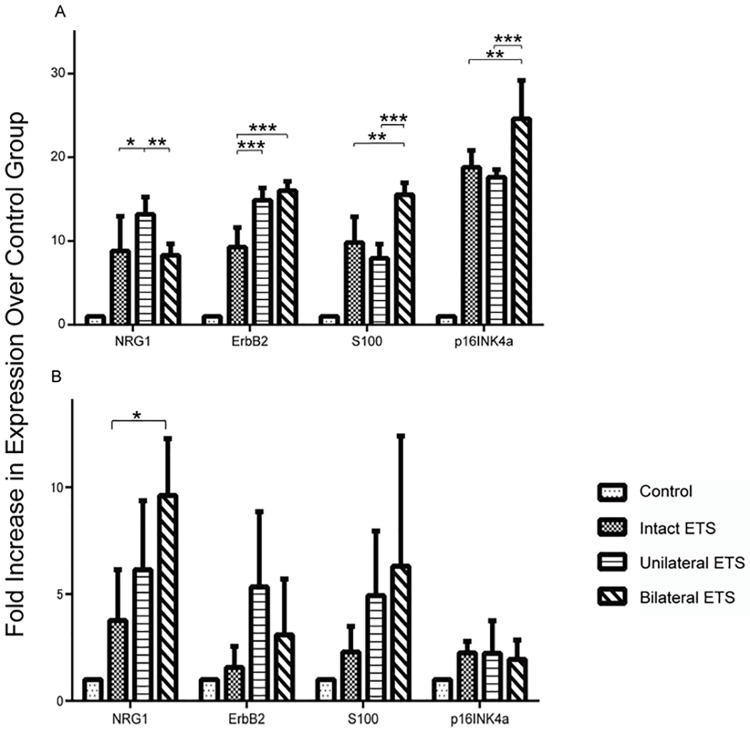
Quantification of Schwann cell migration related gene expression in acellular nerve allografts. A) Two weeks after surgery. The expression of all genes in every end-to-side (ETS) group was upregulated compared with the control group (n = 5 per group). The expressions of S100 and p16^INK4a^ increased the most in the bilateral ETS group. Differences between control and the other groups were significant. B) Four weeks after surgery. The expression of NRG and S100 was the high tendency in the bilateral ETS group, followed by the unilateral and intact ETS groups (n = 5 per group). The expression of p16^INK4a^ dramatically decreased in all groups. Significant differences were only observed between the intact ETS and bilateral ETS group in NRG1. *p < 0.05, **p < 0.01, ***p < 0.001.

At 2 weeks postoperatively, all markers were upregulated. In particular, in the unilateral ETS group, high expression levels of both NRG1 and ErbB2 were noted, and in the bilateral ETS group, high expression level of S100 was observed (p < 0.01). p16^INK4a^ in all groups increased over 17-fold, and the increase was most significant in the bilateral ETS group (p < 0.01) (Fig 5A). At 4 weeks postoperatively, the expression level of ErbB2 in the unilateral ETS group remained higher than that in the other groups. In the bilateral ETS group, the pattern with expression levels of NRG1 and S100 were high and that of ErbB2 were low. In contrast, the upregulation of senescence marker (p16^INK4a^) of SCs was minimal, and low expression pattern of S100, NRG1, and ErbB2 were noted in the intact ETS group (Fig 5B).

### In vivo imaging of axonal elongation

**Fig 6:**
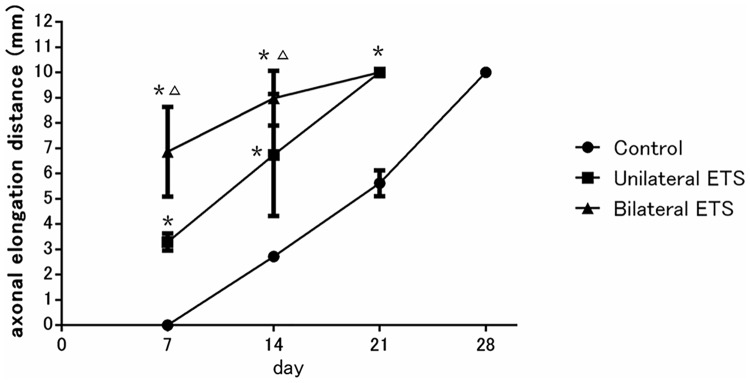
In vivo imaging of axonal elongation in engraftment model. Yellow fluorescent protein-labeled axons entering the acellular nerve allograft (ANG) engraftment were measured every 7 days (n = 4 per group). In particular, axonal elongation accelerated in the bilateral end-to-side (ETS) group at 7 days after surgery. Subsequently, axonal elongation proportionately increased and was achieved at the distal edge of ANG at 21days post transplantation in the unilateral and bilateral ETS groups. Differences between the ANG containing Schwann cell group and control group were significant. *Significantly different from the control group (p < 0.05); Δ, significantly different from the unilateral ETS group (p < 0.05).

GFP expression from regenerated axons was observed in the graft from 7 to 28 days via live imaging (Fig 6). The average axonal elongation from the edge of the graft at 7 days post engraftment was longest in the bilateral ETS group (6.86 ± 1.45 mm), followed by the unilateral ETS (3.29 ± 0.29 mm) and control (0 mm) groups. Fourteen days post engraftment, it was 8.98 ± 0.89 mm in the bilateral ETS group, followed by 5.66 ± 2.09 mm in the unilateral group and 2.72 ± 0.12 mm in the control group. Significant differences were confirmed between unilateral ETS group and bilateral ETS group until 14 days after surgery (p < 0.05). On the 21^st^ day after engraftment, axonal elongation to the distal edge of the graft was completed in the bilateral and unilateral ETS groups. There were significant differences between the ANG containing SC group and control group until 21 days after transplantation (p < 0.05). In the control group, axons reached the distal suture site at 28 days after engraftment.

### Histomorphometry and assessment of nerve cross sections

**Fig 7:**
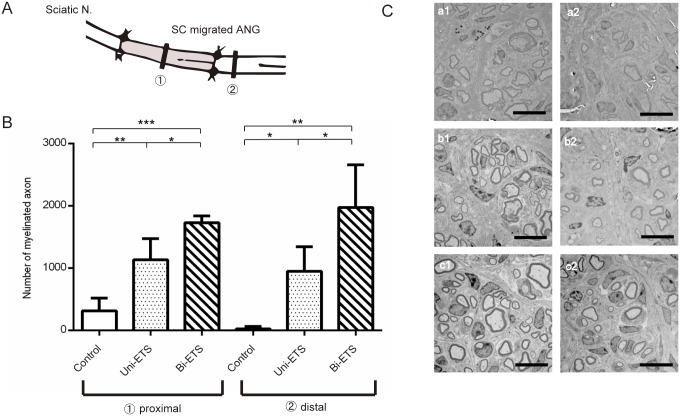
Histomorphometry in the engraftment model. A) Axonal regeneration was evaluated at the 1) proximal: midgraft and 2) distal section, 2 mm distal to the graft (4 weeks post transplantation; n = 4 per group). B) Number of myelinated axon into the graft. Left: proximal, right: distal. Myelinated fibers in the bilateral end-to-side (ETS) group were the highest, followed by the unilateral ETS and control groups. Differences among all groups were significant. *p < 0.05, **p < 0.01, ***p < 0.001. C) Electron micrograph of the proximal and distal section. a1–a2) In the control group, proximal and distal sections, respectively, from regenerating nerves of the control group. A small minority of regenerated fibers were confirmed compared with the other groups. Moreover, histological feature was a predominance of thin myelinated fibers in the three groups. b1–b2) In the unilateral end-to-side (ETS) group, proximal and distal sections, respectively, from regenerating nerves of the unilateral ETS group. The number of regenerated fibers was increased more than the control group. The number of regenerated fibers both proximal and distal section exponentially increased compared with the control group. In addition, there were a number of thick myelinated fibers at the distal section compared with the control group. c1–c2) In the bilateral ETS group, proximal and distal sections, respectively, from regenerating nerves of the bilateral ETS group. The number of regenerated fibers at the distal section dramatically increased compared with the unilateral ETS group. Furthermore, at the distal section, abundant myelinated fibers appeared to have a larger myelin thickness than the other groups. Scale bar, 10 μm.

At the proximal sections (Fig 7A), the total number of regenerating myelinated axons was confirmed as 341.8 ± 242.2 axons in the control group (Fig 7B, a1), 1,174.2 ± 468.7 axons in the unilateral ETS group (Fig 7B, b1), and 1,689.2 ± 616.9 axons in the bilateral ETS group (Fig 7B, c1). The total number of regenerating myelinated axons of the bilateral ETS group was larger than that of unilateral ETS group (p < 0.05) and control (p < 0.001). The control was smaller than the unilateral ETS group (p < 0.01). Furthermore, when we examined the distal sections in the same manner (Fig 7A), 158 ± 282.3 axons in the control group (Fig 7B, a2), 956.2 ± 550.6 axons in the unilateral ETS group (Fig 7B, b2), and 1,434 ± 814.6 axons in the bilateral ETS group were counted (Fig 7B, c2). We confirmed significant differences among all groups at both sections (p < 0.05).

### Quantitative assessment of regeneration and morphometric assessment

**Fig 8:**
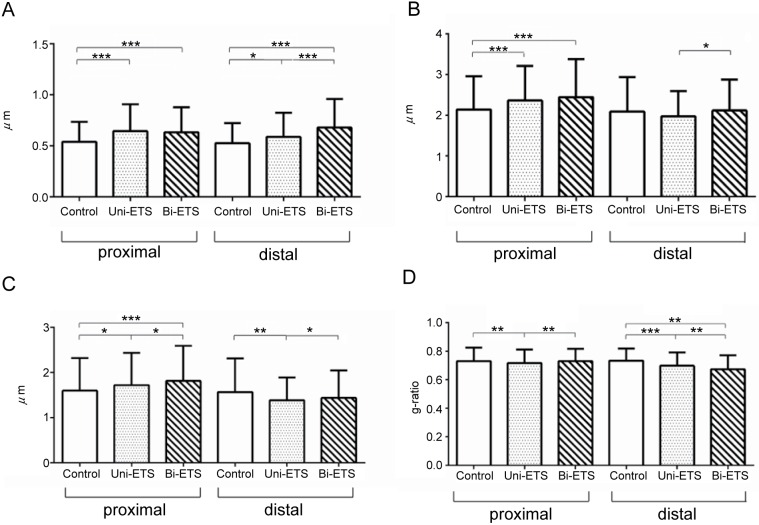
The morphometric parameters of regenerating axons. A) Myelin sheath in acellular nerve allografts containing migrated Schwann cells was thicker than that in the control group. Particularly, differences at distal section among all groups were significant. B) Myelinated fiber diameter at proximal section in the bilateral end-to-side (ETS) group was the largest, followed by the unilateral ETS and control groups. C) Axon diameter showed similar tendency to myelinated fiber diameter at both sections. D) G-ratio at proximal section showed differences among the control, unilateral ETS, and bilateral ETS groups. In contrast, g-ratio at distal section revealed differences among all groups. *p < 0.05, **p < 0.01, ***p < 0.001.

The average myelin sheath diameter of unilateral (0.64 ± 0.26 μm, p < 0.001) and bilateral ETS group (0.63 ± 0.24 μm, p < 0.001) were larger than that of the control group at the proximal portion(0.54 ± 0.19 μm). Similarly, at the distal portions, both the unilateral ETS (0.59 ± 0.24 μm, p < 0.05) and bilateral ETS groups (0.68 ± 0.28 μm, p < 0.001) were larger than the control (0.53 ± 0.19 μm). In addition, at the distal portion, significant differences were observed among all groups (p < 0.05). However, the difference of average myelin sheath thickness was not apparent between the unilateral and bilateral ETS groups at the proximal portion (Fig 8A). At the proximal section of the nerve lesion, the average myelinated fiber diameters of the unilateral (2.37 ± 0.85 μm) and bilateral ETS groups (2.45 ± 0.93 μm) (ANG containing migrated SCs groups) were larger than that of the control group (2.14 ± 0.82 μm, p < 0.001). However, at the distal section, the difference was not apparent between the control and ANG containing migrated SCs groups (Fig 8B). The smaller g-ratio value in the bilateral ETS group showed a greater degree of the myelinated fiber maturation in these groups [38], and the g-ratio of the unilateral ETS group (0.71 ± 0.09) was smaller than that of the control (0.73 ± 0.09, p < 0.01) and bilateral ETS groups (0.73 ± 0.09, p < 0.01) at proximal sections. At the distal section, the g-ratio of the bilateral ETS group (0.67 ± 0.04) was smaller than that of the control (0.73 ± 0.04, p < 0.01) and unilateral ETS groups (0.70 ± 0.04, p < 0.01) (Fig 8D).

### Confocal imaging of neuromuscular junctions

**Fig 9:**
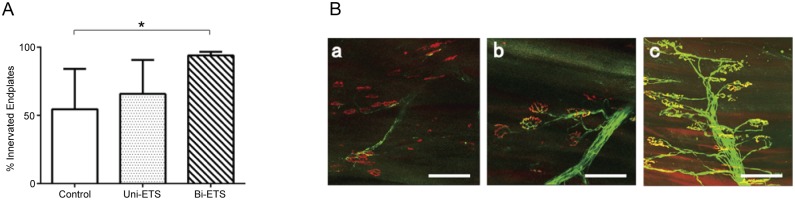
Motor reinnervation at Extensor digitorum longus (EDL) muscle. A) Percentage of reinnervated endplate at 7 weeks after surgery (n = 4 per group). Quantitative analysis of endplates within 25 random samples in Thy1-yellow fluorescent protein 16 mice revealed that reinnervation was significantly better in the bilateral end-to-side (ETS) group than in the other groups (*p < 0.05). B) Whole mount imaging of EDL muscle. a) In the control group, although the majority of motor endplates remained denerved, a few regenerating axons were noted around the endplate. b) The unilateral ETS group showed that a part of endplates were reinnervated. c) The bilateral ETS group demonstrated that most of the endplates were reinnervated and regenerated axons were well organized. Scale bar, 100 μm.

To confirm that axons regenerating across the graft were reinnervating their targets, we quantified the percentage of reinnervated motor endplates at 7 weeks (Fig 9A). Reinnervation of the endplates of EDL muscle was significantly better in the bilateral ETS group where 94% of labeled endplates were reinnervated as compared with 54.5% in the control and 65.8% in the unilateral ETS groups (Fig 9B). Significant differences were only confirmed between the bilateral ETS group and the control (p < 0.05).

## Discussion

Peripheral nerve regeneration with acellular nerve products has not been successful in restoring functional recovery to a similar level as autograft, particularly when gap distances are increased. Numerous investigators have indicated that this is because of the lack of viable SCs that promote axonal regeneration and extra supplementation, such as cultured SCs, neurotrophic factors, or some types of stem cells [16]. However, culturing human SCs in vitro to obtain a sufficient number of viable cells and transplanting these cultured cells to improve axonal regeneration is a time-consuming process [17]. Moreover, human SCs must be derived from autologous sources to avoid immunosuppression and require invasive nerve biopsies, thereby sacrificing healthy nerve tissue [17]. Stem cell therapy is promising but has serious disadvantages, including teratoma formation, potential mislocalization/misdifferentiation, and host immune system response [18, 19, 40]. Most neurotrophic factors, including NGF and BDNF, have a short effective time; thus, continuous growth factor administration is impractical [41].

In this study, we demonstrated that ETS neurorrhaphy, which is used as an axonal provider, could be useful as an SC provider to support axonal elongation in a ANGs *in vivo* model. Our results support the assertion that ETS neurorrhaphy requires an epineurial window to promote the migration of SCs into ANGs; additional partial neurectomy promotes further migration of SCs. Moreover, bilateral ETS neurorrhaphy can accelerate the migration of SCs from both proximal and distal coaptation sites over short times; the number of migrated SCs is larger than that of unilateral SCs. When we transplanted hybrid ANGs containing migrated SCs in the nerve gap, hybrid ANGs with bilateral ETS neurorrhaphy significantly promoted the number of regenerated axons and the distance of axonal elongation as compared with the other groups.

After nerve injury, SCs generally dedifferentiate into immature state, proliferate, and align themselves in the remaining basal lamina to guide regenerating axons to their distal targets [42, 43]. Neuregulin–ErbB signaling is essential for the direct migration of SCs [42, 44]. This study on SC migration ability into ANGs showed that through intact ETS neurorrhaphy, a few SCs migrated only to the proximate coaptation site, and epineurium appeared to interfere with the perforation of SCs into the recipient nerve. Our previous study suggested that even with intact ETS coaptation, sensory axons could regenerate in a delayed fashion through epineurium [23, 26]; however, the creation of an epineural window appeared to be a crucial step for the migration of SCs into entire ANGs. The disruption of the blood–nerve barrier on the perineurium appeared to result in increasing nerve regeneration, with larger perineurial window size corresponding to increased nerve fiber density and the total number of nerve fibers [24]. Those evidences also refer to the importance of epineural window for SC migration into ANGs. Regarding the partial neurectomy to the donor nerve, numerous studies demonstrated that the epineurial window combined with a partial neurectomy of the donor nerve promoted abundant axonal regeneration as compared with the epineurial window without partial neurectomy. Brenner et al. [25] reported this as neurectomy corresponding to approximately one-third of transections resulting in markedly enhanced motor neural regeneration and functional recovery. A total of 40% partial neurectomy with the epineurial window showed good reinnervation of the recipient nerve without structural and functional changes of the donor system during a long-term evaluation [28, 45]; dose-response relationships between the axotomy of the donor nerve and donor nerve regeneration has previously been demonstrated [25, 45]. In our unilateral ETS neurorrhaphy group, which was transected approximately one-fifth, revealed the rapid migration of SCs into ANGs as compared with the intact ETS neurorrhaphy group. *In vivo* imaging of the SC migration in the axotomy models, irrespective of it being unilateral or bilateral, showed a similar migration distance at 2 weeks after surgery. Therefore, we suspected that axotomy is one of the important factors to accelerate the migration of SCs into the entire graft. Since the higher expression of NRG1 and ErbB2 in the unilateral ETS group also supports this theory at 4 weeks after surgery, we inferred that the migration of SCs had persisted. Regarding bilateral ETS coaptation, our previous study demonstrated that SCs migrate equally into ANG from both proximal and distal coaptation sites, and in case grafted SCs existed, host SCs did not migrate into the graft [31]. In this study, the bilateral ETS neurorrhaphy group up-regulated expression of S100 from at 2 weeks after surgery, and we could observe comparable migration of SCs from both proximal and distal coaptation through live imaging. The high expression of ErbB2 at 2 weeks after surgery implied immature SC and S100, thereby indicating that a certain amount of SCs had already migrated. After 4 weeks after surgery, as for the high expression level of neuregulin-1 and the decrease of ErbB2 expression level, we could speculate that the migration of SCs had already completed, and SCs got matured with considerable axonal regeneration. The level of S100 was higher than that of the other groups; bilateral ETS effectively increased the amount of migrated SCs from both anastomoses.

In contrast, Saheb-Al-Zamani et al. [46] recently reported that SCs migrated into long ANG and were led into senescence and limited axonal regeneration into the graft as gap length became longer. The mechanism of senescence remained unclear; however, they suspected that excessive proliferative demand on donor SCs to populate increasing acellular volumes or prolonged absence of trophic support from axons because of chronic denervation could contribute to senescence. Poppler et al. [47] recently reported that long ANGs were repopulated with increased p16-positive SCs and stromal cells compared to short ANGs, suggesting that the ratio of SCs expressing markers of senescence (p16) was directly related to a poor regenerative outcome across nerve constructs.

Even with the evidence of SC senescence, ETS neurorrhaphy effectively induced the migration of SCs into ANGs, and those SCs facilitated considerable axonal regeneration. Saheb-Al-Zamani et al. [46] and Poppler et al.[47] evaluated SC senescence from the level of p16^INK4a^, and in our study, the level of p16^INK4a^ was up-regulated in all ETS neurorrhaphy groups at 2 weeks after surgery and significantly decreased at 4 weeks after surgery. It may imply that early excessive stress for SC migration through ETS neurorrhaphy induced upregulation of p16^INK4a^ and induce migrating SCs into senescence. However, all ETS groups showed same tendency, and the upregulation of p16^INK4a^ was observed only at early after surgery. P16^INK4a^ is also activated through the Ras-Raf-Mek pathway, a pathway for SC proliferation [48–50]. Thus, the upregulation of p16^INK4a^ may result from abundant SC proliferation. However, there was no relevant evidence for SC senescence up to 4 weeks of SC induction through ETS neurorrhaphy. When we applied ETS bilaterally to the ANG, the axons regenerated into the ANG from both ETS anastomoses, and the length of axons, which facilitate SCs, doubled compared with regular nerve graft in which axons are regenerated only from the proximal anastomosis.

Therefore, we hypothesized a few theories for favorable axonal regeneration in our hybrid ANG models through ETS neurorrhaphy. First, we could induce SCs into ANGs through ETS neurorrhaphy without senescence of SCs, and we could increase the amount of SCs by partial neurectomy to the donor nerve and applied ETS neurorrhaphy to both edges of the graft. Second, if SCs were led into senescence at early postoperative stress in 2 weeks period, migrated SCs matured in ANGs by 4 weeks period and provided pro-regenerative environment that facilitates nerve regeneration [14, 51]. Third, bilateral ETS provides better result because increasing the number of anastomosis decreases the distance that each SC required for migration and diminishes the stressful stimuli of migration subsequently.

The efficacy of ETS neurorrhaphy as an axonal provider has been obvious not only *in vivo* but also clinically [29]. It has been utilized in several situations, such as sensory nerve reconstruction for hand or facial reanimation [30, 52, 53]. Functional deficit to the donor nerve has been kept minimal or none; therefore, we could apply this technique as SC provider, relatively soon in adequate clinical situations. However, there are still several issues that we need to elucidate to put this concept into practice. First, we need to evaluate the length of ANGs we could apply this concept to and adequate migration period for each length of ANGs. Second, we need to clarify whether increasing ETS coaptation could reduce stress for migration in SCs and avoid SC senescence. Third, we should evaluate whether SC-migrated hybrid ANGs could be a substitute for autograft. We will attempt to make our study more specific for clinical application and try to make ANGs more practical for a variety of nerve reconstructions.

In conclusion, ETS neurorrhaphy, which is used as an axonal provider, could be useful as an SC provider to support axonal elongation in ANGs *in vivo*. It is effective when an epineurial window and partial neurectomy is performed and is more effective when applied bilaterally. This approach can potentially represent a new usage of ETS neurorrhaphy and make ANGs more practical by creating hybrid nerve graft in which autologous SCs are combined.
